# Extracellular vesicles from a novel *Lactiplantibacillus plantarum* strain suppress inflammation and promote M2 macrophage polarization

**DOI:** 10.3389/fimmu.2024.1459213

**Published:** 2024-08-23

**Authors:** Shuang Gong, Ruixia Zeng, Ling Liu, Rui Wang, Man Xue, Hao Dong, Zhigang Wu, Yibo Zhang

**Affiliations:** ^1^ School of Basic Medical Sciences, Jinzhou Medical University, Jinzhou, Liaoning, China; ^2^ Department of Human Anatomy, School of Basic Medical Sciences, Jinzhou Medical University, Jinzhou, Liaoning, China; ^3^ Department of Pathogenic Microbiology, School of Basic Medical Sciences, Jinzhou Medical University, Jinzhou, Liaoning, China; ^4^ Collaborative Innovation Center for Prevention and Control of Zoonoses, Jinzhou Medical University, Jinzhou, Liaoning, China

**Keywords:** *Lactiplantibacillus plantarum*, extracellular vesicles, lipopolysaccharide, macrophages, anti-inflammatory

## Abstract

**Background:**

*Lactiplantibacillus plantarum* (*L. plantarum)* is known for its probiotic properties, including antioxidant and anti-inflammatory effects. Recent studies have highlighted the role of extracellular vesicles (EVs) from prokaryotic cells in anti-inflammatory effects.

**Objective:**

This study aims to investigate the anti-inflammatory effects of extracellular vesicles derived from a newly isolated strain of *L. plantarum* (LP25 strain) and their role in macrophage polarization.

**Methods:**

The LP25 strain and its extracellular vesicles were isolated and identified through genomic sequencing, transmission electron microscopy (TEM), and nanoparticle tracking analysis (NTA). RAW 264.7 cells were treated with lipopolysaccharide (LPS) and/or LP25-derived extracellular vesicles (LEV). Morphological changes in the cells were observed, and the expression levels of pro-inflammatory cytokines (TNF-α, IL-6)、iNOS and anti-inflammatory cytokines (IL-10) 、Arg-1 were measured using quantitative real-time PCR (qPCR). Flow cytometry was used to detect the expression of Arg-1 in the treated cells.

**Results:**

Treatment with LP25 EVs led to significant morphological changes in RAW 264.7 cells exposed to LPS. LP25 EVs treatment resulted in increased expression of Arg-1 and anti-inflammatory cytokines IL-10, and decreased expression of iNOS and surface markers protein CD86. Flow cytometry confirmed the increased expression of the M2 macrophage marker Arg-1 in the LP25 EVs-treated group.

**Conclusion:**

Extracellular vesicles from *Lactiplantibacillus plantarum* LP25 can suppress inflammatory responses and promote the polarization of macrophages toward the anti-inflammatory M2 phenotype. These findings provide new evidence supporting the anti-inflammatory activity of *L. plantarum*-derived EVs.

## 1 Introduction

*L. plantarum* is a type of Gram-positive bacterium widely found in fermented foods, with a long history of safe consumption. Most strains of *L. plantarum* have been demonstrated to possess probiotic behaviors and functions, including antioxidant and anti-inflammatory activities (1, 2), as well as various physiological functions such as inhibiting intestinal pathogens, improving obesity, and regulating gut microbiota (1). Recent studies have shown that EVs from Gram-positive bacteria play a crucial role in health and disease (3), although Gram-positive bacteria were previously thought incapable of producing EVs due to their thick peptidoglycan cell wall.

Extracellular vesicles (EVs) are nanoscale vesicular structures secreted by both bacterial and eukaryotic cells, generated through the budding of cell membranes or organelle membranes (4). These vesicles possess a lipid bilayer membrane structure and encapsulate a variety of biomolecules including DNA, RNA, proteins, polysaccharides, lipids, various metabolites, and signaling molecules. The composition of EVs is complex, and their biological functions generally vary depending on the producing organism (5). Recently, EVs from *L. plantarum* APsulloc 331261, originally derived from green tea leaves, have been shown to trigger M2 macrophage polarization and induce an anti-inflammatory response in human skin (6). However, not all strains of *L. plantarum* exhibit anti-inflammatory effects and induce M2 macrophage polarization.

Macrophages are crucial host immune cells that play a central role in initiating and regulating inflammatory responses (7). Depending on local microenvironmental stimuli, macrophages can polarize into pro-inflammatory M1 and anti-inflammatory M2 phenotypes, which respectively trigger and suppress host inflammatory responses (8). M1 macrophages are primarily stimulated by interferon-γ (IFN-γ), lipopolysaccharides (LPS), and tumor necrosis factor-α (TNF-α). They secrete pro-inflammatory cytokines (TNF-α, IL-6, IL-1β), promote the production of monocyte chemoattractant protein-1 (MCP-1) and inducible nitric oxide synthase (iNOS), and express surface markers such as CD86. These molecules’ functions contribute to the development of inflammation, extracellular matrix degradation, cell apoptosis, and regulation of Th1-type immune responses (9, 10). Conversely, M2 macrophages are mainly stimulated by macrophage colony-stimulating factor (MCSF), IL-4, and IL-13. They secrete anti-inflammatory cytokines (IL-10), transforming growth factor-β (TGF-β), express arginase-1 (Arg-1), and promote the expression of the surface marker Arg-1. M2 macrophages inhibit T cell proliferation and activation, regulate Th2-type immune responses, and facilitate tissue remodeling (7). Notably, M1 and M2 macrophages can undergo reversible transformations under certain conditions (11). Therefore, effectively regulating monocyte/macrophage polarization is key to inhibiting inflammatory responses and promoting repair. Thus*, L. plantarum* strains that produce EVs promoting M2 macrophage polarization or reducing pro-inflammatory M1 expression are potential probiotics with anti-inflammatory effects.

In this study, we collected locally fermented dairy products, specifically milk tofu, from Inner Mongolia Autonomous Region, China. These samples were then transported to Jinzhou Medical University, Liaoning Province, through a cold chain. We screened and identified a strain of *L. plantarum* LP25 by analyzing the morphology of RAW 264.7 macrophages and the levels of the M2 macrophage-associated factor IL-10 induced by bacterial supernatants from 31 strains isolated from dairy products in Inner Mongolia, China. Subsequently, we isolated extracellular vesicles (EVs) from this strain and found that they can inhibit LPS-induced M1 polarization of RAW 264.7 macrophages and induce M2 polarization. Additionally, the effect of these EVs on macrophage M2 polarization was demonstrated through *in vitro* experiments.

## 2 Methods

### 2.1 Isolation and cultivation of *Lactiplantibacillus plantarum*

Fermented dairy products were collected locally, specifically milk tofu, from Inner Mongolia Autonomous Region, China. These samples were then transported to Jinzhou Medical University in Liaoning Province via cold chain logistics. Using MRS agar medium (Qingdao Hope Bio-Technology Co., Ltd., China), which consists of 10 g/L proteose peptone, 8 g/L beef extract, 4 g/L yeast extract, 20 g/L glucose, 2 g/L dipotassium phosphate, 2 g/L diammonium hydrogen citrate, 5 g/L sodium acetate, 0.2 g/L magnesium sulfate, 0.04 g/L manganese sulfate, 14 g/L agar, and 1 g/L Tween 80, 31 strains of *Lactobacillus plantarum* were isolated. All strains were stored in 25% glycerol at -80°C. A bacterial suspension with a concentration of 10^9^ CFU/mL was prepared and inoculated into 10 mL of MRS broth (Qingdao Hope Bio-Technology Co., Ltd., China), which has the same composition as the MRS agar medium but without the agar. The cultures were incubated at 37°C with shaking at 200 r/min for 72 hours. After incubation, the cultures were centrifuged at 4000 r/min at 4°C for 10 minutes. The supernatant was then filtered through a 0.22 µm sterile membrane filter, and the filtered supernatant was aliquoted and stored at -80°C.

BNCC299253, *Lactiplantibacillus plantarum* (Orla-Jensen) Bergey et al. (American Type Culture Collection), was cultured in same condition with the isolated strains.

### 2.2 Cell culture

RAW 264.7 murine macrophages were obtained from Meilun Biotechnology Co., Ltd. (Suzhou, China). The RAW 264.7 cells were cultured in high-glucose DMEM(Gibco)supplemented with 10% FBS (Zhejiang Tianhang Biotechnology Co., Ltd., China), 100 U/mL penicillin, and 100 μg/mL streptomycin (Caisson Labs, USA) at 37°C in a humidified incubator with 5% CO2. The cells were treated with supernatant (10μg/ml) of each of the 31 strains for 18 hours, cell morphology was observed using a Leica inverted microscope. Phase-contrast images were taken, and the long and short axes of each cell were measured using ImageJ software. The elongation factor was determined by the ratio of the two axes. Cell area was calculated by outlining the cell contours. To value the anti-inflammatroy effect of the LP25, the cells were treated with *L. plantarum* LP25 extracellular vesicles (LP25 EVs) (240 μg/ml) and LPS (1 μg/mL) (Escherichia coli serotype (O111:B4), Sigma-Aldrich) for 18 hours, with LPS-stimulated RAW 264.7 cells serving as the control.

### 2.3 Isolation of *L. plantarum* LP25 extracellular vesicles

Previous studies have shown that the production of EVs from *L. plantarum* NBRC 15891 varies with cultivation time, reaching the highest concentration at 72 hours (12). Accordingly, in this study, the supernatant of 72-hour cultures was used for LP25 EVs preparation. Following the polyethylene glycol (PEG) precipitation method (13), 10^9^ CFU/mL of bacterial suspension was inoculated into 1 L of MRS broth. The culture was incubated at 37°C with shaking at 200 r/min for 72 hours. The culture was centrifuged at 500 g for 10 minutes at 4°C, and the supernatant was collected and centrifuged again at 2000 g for 30 minutes at 4°C. The supernatant was further centrifuged at 10,000 g for 30 minutes at 4°C and then filtered through a 0.22 μm vacuum filter. PEG-8000 (Sigma-Aldrich) was added to the supernatant to a final concentration of 5% and incubated overnight (at least 12 hours) at 4°C with shaking. The mixture was centrifuged at 10,000 g for 1 hour at 4°C, and the pellet was resuspended in sterile PBS and stored at -80°C. LP25 EVs was characterized using transmission electron microscopy (TEM) and nanoparticle tracking analysis (NTA) at Wuhan MaiSp Biotech Co., Ltd. The protein concentration of LP25 EVs was determined using a BCA Protein Assay Kit (Beyotime Biotechnology Co., Ltd., Shanghai, China.) according to the manufacturer’s instructions.

### 2.4 ELISA

RAW264.7 cells were seeded in 24-well plates at a density of 2 × 10^5^/well. The culture supernatant (10μg/ml) of each of the 31 strains was added for 18 hours, the supernatants were collected and the levels of IL-10 were determined by Mouse Interleukin 10 ELISA Kit (Beijing Chenglin Biotechnology Co., Ltd., China) following the manufacturers’ recommendations and quantified using ALLSHENG (Hangzhou, China) microplate reader at 450 nm immediately, and IL-10 concentrations were calculated based on a standard curve.

### 2.5 Reverse-transcription quantitative polymerase chain reaction (RT-PCR)

RNA was extracted using Trizol reagent (Vazyme). RNA concentration was measured with a UV spectrophotometer (HITACHI). Reverse transcription was performed using the ABScript Neo RT Master Mix kit (ABclonal). Quantitative polymerase chain reaction (qPCR) was carried out using Genious 2X SYBR Green Fast qPCR Mix (ABclonal) and analyzed with a qPCR instrument (Gentier 96, Tianlong). Primers used for qPCR are listed in **Table 1**. Gene expression was analyzed using the 2−ΔΔCt method.

**Table 1:** List of primer pairs used for qRT-PCR.

Gene	Forward (5’-3’)	Reverse (5’-3’)
TNF-α IL-6 iNOS Arg-1 IL-10 β-actin	TATGGCCCAGACCCTCACA GAGGATACCACTCCCAACAGACC AGCGAGGAGCAGGTGGAAGA GTGTACATTGGCTTGCGAGA CGGGAAGACAATAACTGCACCC TCGAGTCGCGTCCACC	GGAGTAGACAAGGTACAACCCATC AAGTGCATCATCGTTGTTCATACA GAGGGGGGAATGACATGAGG GGTCTCTTCCATCACCTTGC CGGTTAGCAGTATGTTGTCCAGC GGGAGCATCGTCGCCC

### 2.6 Western blot

RAW 264.7 macrophages were seeded in 6-well plates at a concentration of 2 × 10^6 cells/mL. After 24 hours, the medium was replaced with serum-free high-glucose DMEM, and the cells were treated with LPS (10 ng/mL) or LPS plus LP25 EVs for 18 hours. To obtain protein samples, cells were lysed in a lysis buffer consisting of NP40 (Solarbio, Beijing, China) and PMSF(100mM)(Phenylmethanesulfonyl fluoride) (Solarbio, Beijing, China)at a ratio of 100:1. Protein concentration was determined using a BCA Protein Assay Kit, and samples were denatured at 100°C for 10 minutes in 5× loading buffer. Proteins were separated by 15% SDS-PAGE and transferred onto PVDF membranes. The membranes were blocked with 5% non-fat milk at room temperature for 2 hours and incubated overnight at 4°C with primary antibodies against rabbit polyclonal antibody to Arg-1(1: 1000), IL-10(1: 1000), TNF-α(1: 500), CD86(1: 1000), and β-actin(1: 3000)(Affinity Biosciences, Cincinnati, USA). After washing with PBST, the membranes were incubated with anti-rabbit secondary antibodies (1: 8000)at room temperature for 2 hours and washed again with PBST. Protein bands were visualized using an enhanced chemiluminescence imaging system (CLiNX, Shanghai, China). Band intensities were quantified using ImageJ software.

### 2.7 Flow cytometry

Six-week-old male C57BL/6 mice (15 ± 2g) were obtained from Changsheng Biotechnology Co. (Liao Ning, China). The mice were allowed to live in controlled environmental conditions (ambient temperature, 23 ± 1°C; relative humidity, 50 ± 5%; 12-h light/dark cycle). The animals were freely allowed drinking water and a standard pelleted diet. All protocols for animal experiments were approved by the Animal Ethics Committee of Jinzhou Medical University (approval ID: 2022030601). Mice were maintained under standard laboratory conditions for one week prior to the experiments and then divided into two groups of 6–8 mice.

C57BL/6 mice received an intraperitoneal injection of 200 μl of LP25 EVs (5 mg/mL). After 8 hours, peritoneal cells were harvested by washing the peritoneal cavity with PBS. The collected cells were then centrifuged at 4°C, 1000 r/min for 3 minutes. Subsequently, the cells were treated with CD16/32 at 4°C for 30 minutes to block Fc receptors(2.4G2,BD Biosciences), followed by incubation with Apc-conjugated anti-mouse CD45.2(104,Biolegend), Apc-cy7-conjugated anti-mouse F4/80(BM8,Biolegend), and percp/cy5.5-conjugated anti-mouse CD11b antibodies(M1/70,Biolegend) or isotype-matched controls on ice for 15 minutes in the dark. Next, 300 μl of fixative (1X) was applied on ice for 30 minutes. This was followed by the addition of 700 μl of membrane breaking solution (1X), centrifugation at 4°C and 2000 r/min for 5 minutes. The cells were then incubated with FITC-conjugated anti-mouse Arg-1 antibody (IC5868F,R&D Systems) and PE-conjugated anti-mouse iNOS antibody (CXNFT, eBioscience) for 1 hour at room temperature. Finally, 1ml of membrane-breaking fluid was vortexed and centrifuged at 4°C, 2000 r/min for 5 minutes. Excess unbound antibodies were removed, and the labeled cells were analyzed using a FACSCanto™ II flow cytometer (BD Biosciences). Data on average fluorescence intensity were processed and quantified with FlowJo 10.8.1 software.

### 2.8 Statistical analysis

Results are presented as the mean ± SD from three independent experiments conducted in triplicate. Differences between conditions were evaluated using one-way ANOVA and two-tailed Student’s t-tests. A p-value of less than 0.05 was considered statistically significant. All calculations were performed using GraphPad Prism 10.0 software.

## 3 Results

### 3.1 Screening of anti-inflammatory *L. plantarum*

**Figure 1:**
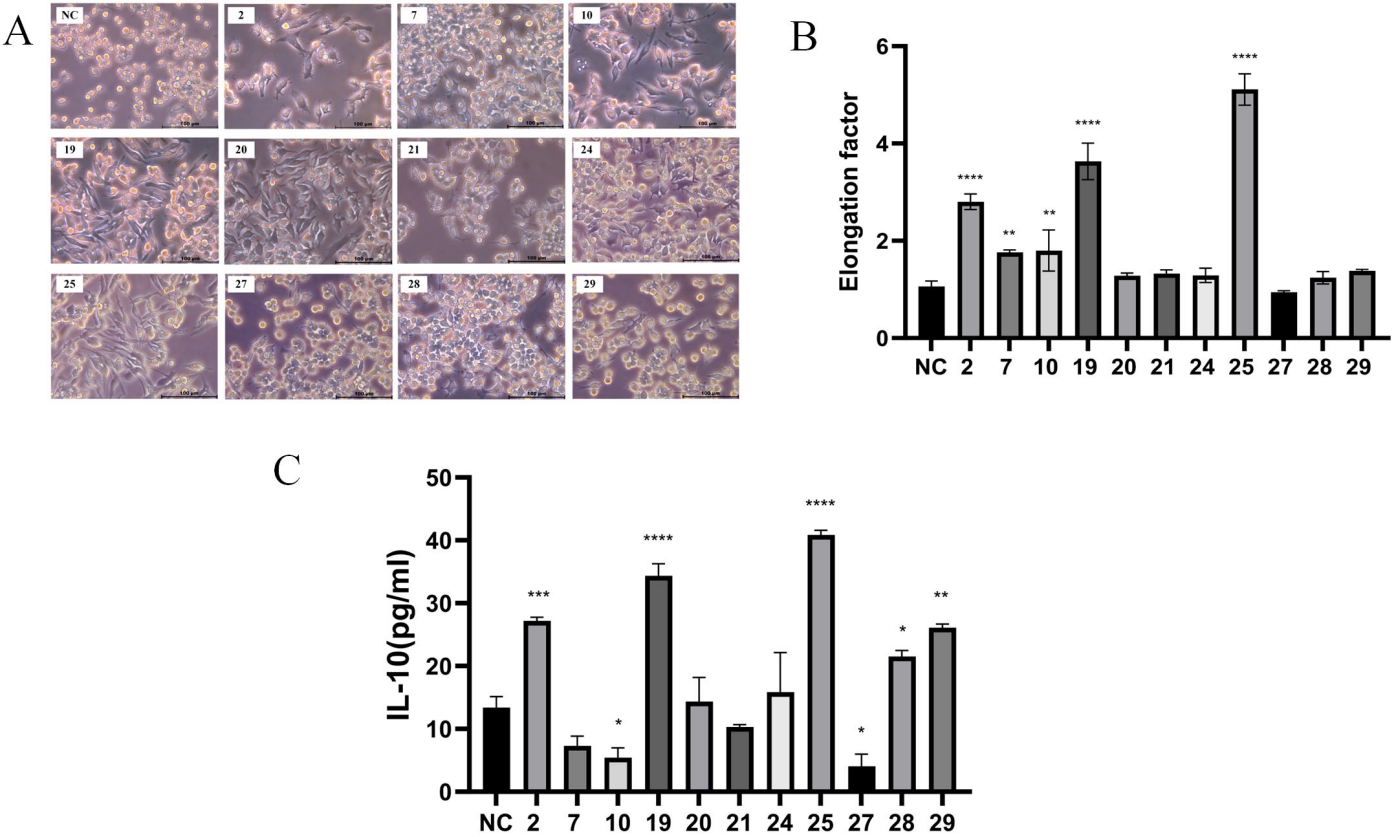
Screening of anti-inflammatory *Lactobacillus plantarum* strains. The cells were treated with supernatant (10μg/ml) of each of the 31 strains for 18 hours, cell morphology was observed using a Leica inverted microscope. **(A)** Images of RAW 264.7 macrophages treated with the supernatant of screened partial bacterial strains (scale bar = 100 μm); **(B)** Quantification of the area for each group; **(C)** ELISA detection of IL-10 concentration after 18 hours of treatment with the partial bacterial supernatant. Error bars indicate mean ± SD for three separate experiments. *p < 0.05, **p < 0.01, ***p < 0.001, ****p < 0.0001 compared with the NC, which indicated as untreated cells.

A total of 31 strains of lactic acid bacteria were isolated from fermented foods using selective MRS agar plates. Previous reports indicated that M2 macrophages exhibit an elongated shape compared to M1 macrophages (14). The morphology of RAW 264.7 macrophages treated with the supernatant from strain 25 was significantly more elongated compared to those treated with other strains (**Figures 1A, B**).To further evaluate the pro-inflammatory or anti-inflammatory effects of the strains, the concentration of IL-10 (an anti-inflammatory cytokine) in the supernatant was measured using ELISA (15). After 18 hours of treatment with the supernatant from strain 25,which is from Inner Mongolia Autonomous Region, China, the most significant changes in cell morphology and IL-10 concentration were observed (**Figure 1C**).

### 3.2 Identification and biological characteristics analysis of *L. plantarum* LP25

**Figure 2:**
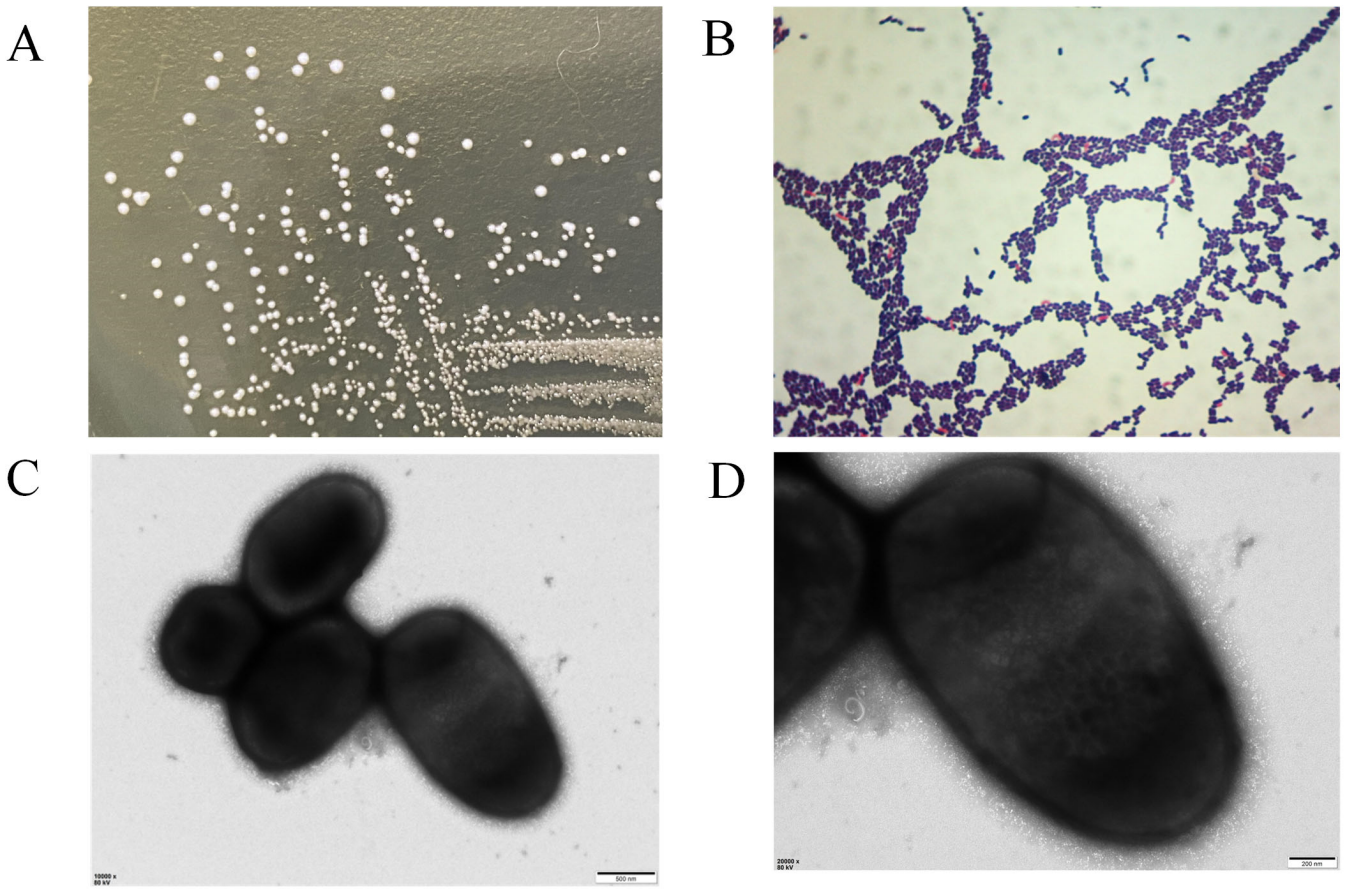
Morphological observation of *Lactobacillus plantarum* LP25. **(A)** Growth morphology of LP25 on MRS agar; **(B)** Gram staining of LP25; **(C, D)** Transmission electron microscopy images of LP25 at 500 nm and 200 nm magnification.

When cultured on MRS agar at 37°C overnight, the colonies of strain LP25 were circular, milky white, smooth, convex, with neat edges, and opaque. Gram staining showed that the strain was Gram-positive (**Figures 2A, B**). The bacterial suspension was examined under a transmission electron microscope at Wuhan MaiSp Biotech Co., Ltd., revealing short, broad rod-shaped cells without pili or flagella (**Figures 2C, D**).

The growth curve of strain LP25 showed that the bacteria entered the stationary phase at 12 hours (**Supplementary Figure S1A**). The strain can grow in a microaerophilic environment with 5.0% CO2 and also thrives under both anaerobic and aerobic conditions. Furthermore, it can grow in a pH range of 2.0 to 10.0, with optimal growth at pH 7.0 (**Supplementary Figure S1B**). Additionally, the strain can grow at temperatures ranging from 10 to 45°C, with optimal growth at 37°C (**Supplementary Figure S1C**). Moreover, it can grow in salt concentrations up to 5%, with optimal growth at 0% salt (**Supplementary Figure S1D**). Finally, the 16S rRNA gene tree and core genome tree of the strain indicate that it is a strain of L. plantarum (**Supplementary Figures S1E, F**).

### 3.3 Reduced expression of M1 macrophage markers in LPS-stimulated RAW264.7 cells treated with LP25 EVs

**Figure 3:**
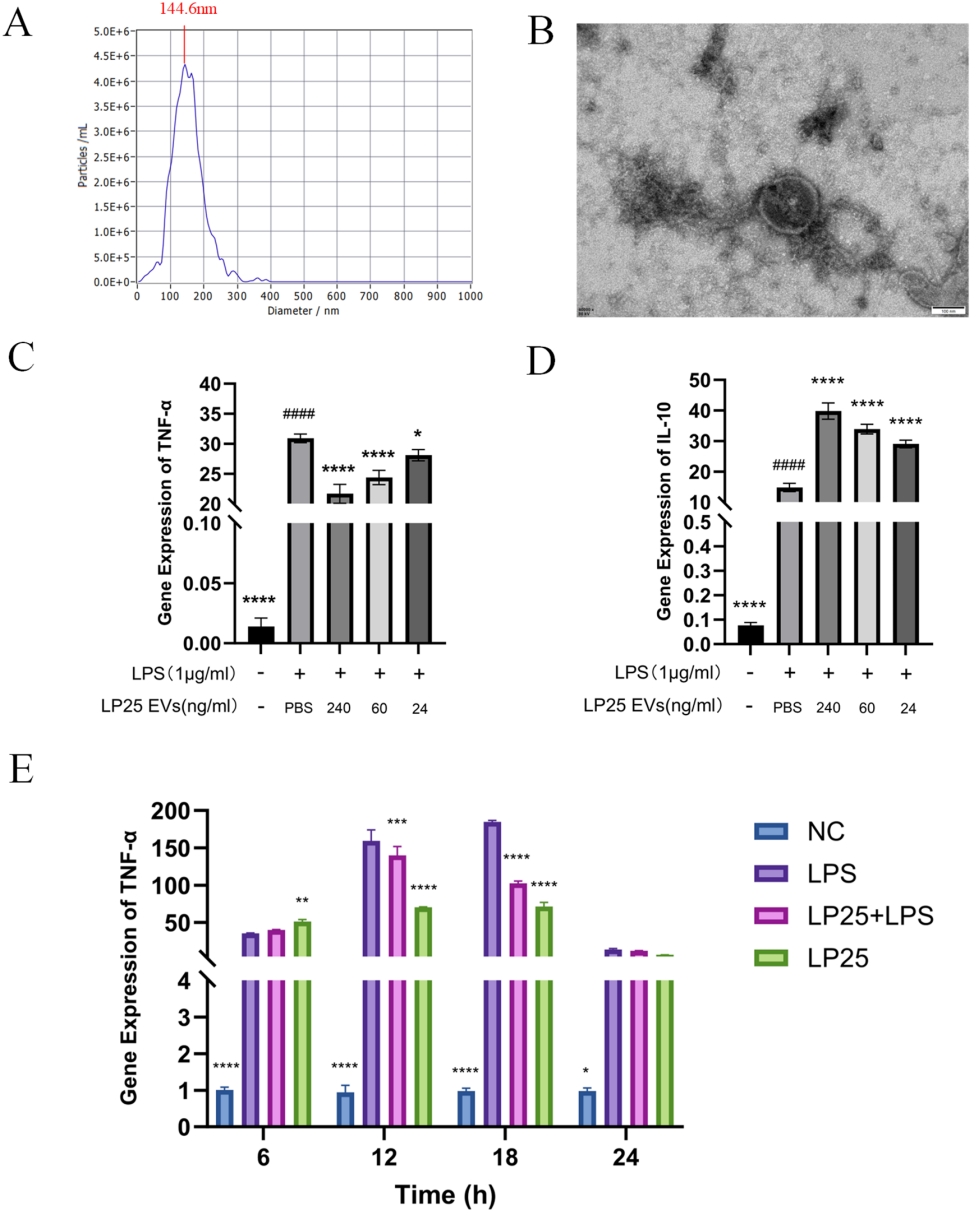
Identification of LP25 extracellular vesicles and expression levels of TNF-α and IL-10 at different time points or concentrations. 10^9^ CFU/mL of bacterial suspension was inoculated into 1 L of MRS broth. The culture was incubated at 37tiowith shaking at 200 r/min for 72 hours, LP25 EVs were isolated from the supernatants by PEG precipitation method. **(A)** Nanoparticle tracking analysis (NTA) of LP25 extracellular vesicles; **(B)** Transmission electron microscopy (TEM) image of LP25 extracellular vesicles; **(C, D)** At 18 hours post-treatment, qPCR analysis was conducted on LPS-induced RAW 264.7 macrophages treated with LEV at concentrations of 240 ng/ml, 60 ng/ml, and 24 ng/ml to assess the expression levels of the relevant genes TNF-α and IL-10; **(E)** qPCR detection of M1 macrophage phenotype-related genes TNF-α expression after 6 hours,12 hours,18 hours and 24 hours of LPS(1μg/ml) treatment or LPS(1μg/ml)/LEV (240 ng/ml) treatment. Error bars represent mean ± SD from three independent experiments. ^####^ *p*<0.0001 indicates a significant difference from PBS group; *p<0.05, **p<0.01, ***p<0.001, ****p<0.0001 indicate significant differences from the LPS-treated group.

To assess if the anti-inflammatory effects in the bacterial supernatant are linked to extracellular vesicles (EVs), we extracted EVs from the LP25 strain. The size of the extracellular vesicles (EVs) was determined using both Transmission Electron Microscopy (TEM) and Nanoparticle Tracking Analysis (NTA). NTA offered a comprehensive size distribution analysis, reporting a median particle size (X50) of 145.8 nm and a peak size around 144.6 nm (**Figure 3A**). TEM provided detailed images showing the most frequent particle size, approximately 150 nm (**Figure 3B**).

Quantitative analysis of TNF-α and IL-10 expression at different LP25 EVs concentrations was performed using qPCR, as shown in **Figures 3C, D**. The concentration titration of LP25 EVs revealed a notable decrease in TNF-α expression at 240 ng/ml (**Figure 3C**), along with an increase in IL-10 expression (**Figure 3D**). Subsequent the results indicated a significant inhibition of pro-inflammatory cytokine expression by LP25 EVs after 18 hours of LPS induction (**Figure 3E**). Following this, a LP25 EVs concentration of 240 ng/ml was used for an 18-hour culture period to perform subsequent experiments.

Additionally, we performed apoptosis assays using a one-step TUNEL apoptosis detection kit (Biyuntian, China) to compare with *Lactobacillus plantarum* 299253. The results showed that LP25 EVs did not induce apoptosis in RAW 264.7 macrophages and simultaneously reduced LPS-induced apoptosis (**Supplementary Figure S2**).

**Figure 4:**
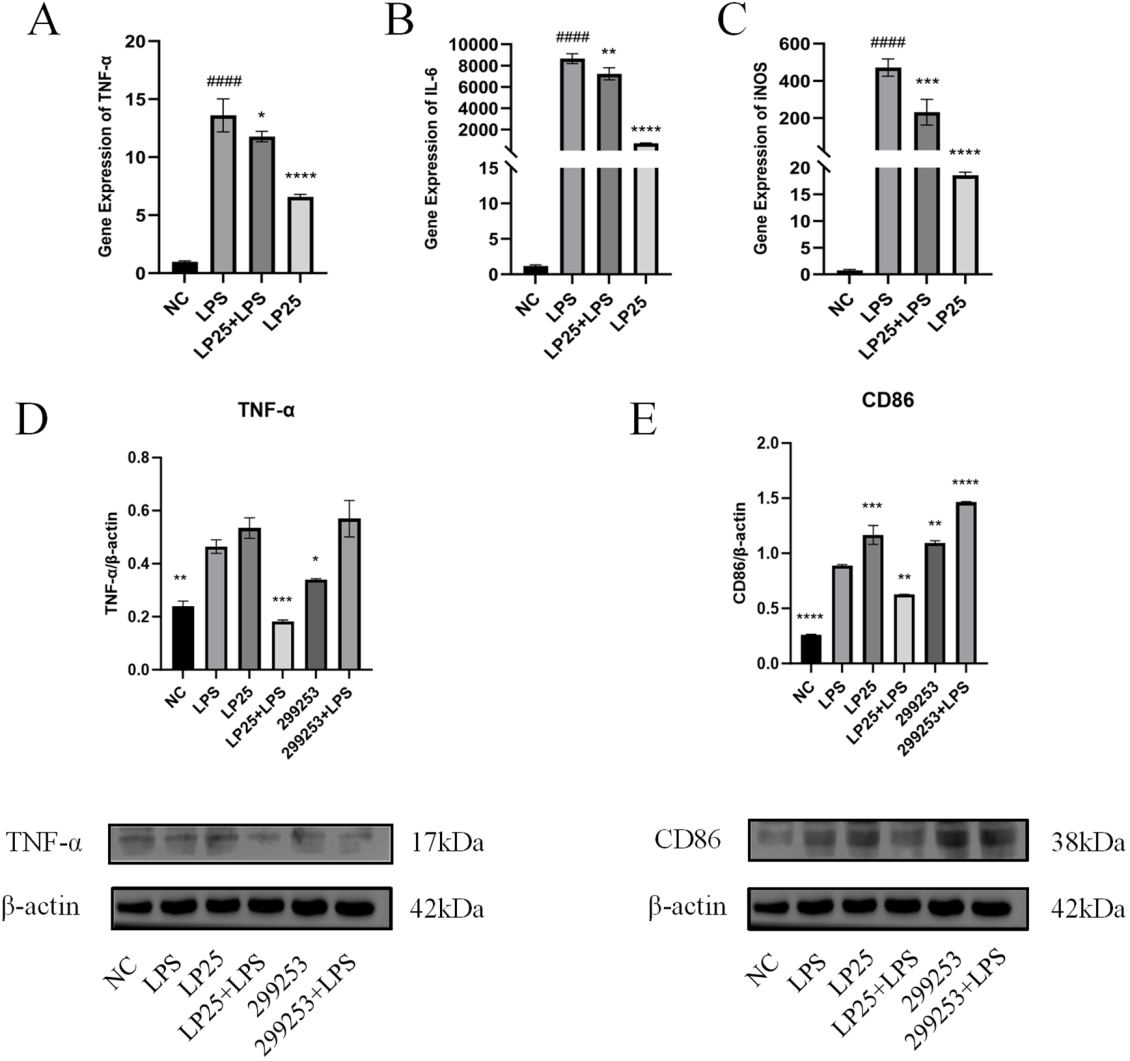
Expression of M1 macrophage markers in LPS-stimulated RAW 264.7 cells treated with LP25 EVs. **(A–C)** qPCR detection of M1 macrophage phenotype-related genes TNF-α、IL-6 and iNOS expression after 18 hours of LPS (1μg/ml) treatment or LPS (1μg/ml)/LP25 EVs (240 ng/ml) treatment; **(D, E)** Representative Western blot and average quantitative data from three independent experiments of TNF-α, CD86, and β-actin expression after 18 hours of LPS(1μg/ml) treatment or LPS/LP25 EVs treatment. Error bars represent mean ± SD from three independent experiments. ^####^ *p*<0.0001 indicates a significant difference from NC,NC indicate LPS-untreated cells; *p < 0.05,**p < 0.01, ***p < 0.001, ****p < 0.0001 indicate significant differences from the LPS alone.

To investigate whether LP25 EVs could modulate M1 polarization in LPS-stimulated RAW264.7 cells (1 μg/mL), we used quantitative PCR (qPCR) to assess mRNA expression of different macrophage phenotypes and Western blotting to evaluate protein expression. LP25 EVs treatment (240 μg/mL) for 18 hours inhibited the expression of M1 phenotype markers TNF-α, IL-6, and iNOS in LPS-stimulated RAW 264.7 cells (**Figures 4A–C**). As shown in **Figures 4D, E**, LP25 EVs from the standard *L. plantarum* strain BNCC 299253 were used as a control. The expression of M1 phenotype markers TNF-α and surface markers protein CD86 was reduced in LPS-stimulated RAW 264.7 cells treated with LP25 EVs, but the 299253 strain did not exhibit the same trend.

Currently, ultracentrifugation is regarded as the “gold standard” for exosome isolation (13). Therefore, we compared LP25 EVs obtained by PEG precipitation with those obtained by ultracentrifugation. LP25 EVs (240 μg/mL) were used to treat LPS-stimulated RAW 264.7 macrophages, and the expression of the M2 polarization marker Arg-1 protein was assessed. No significant differences between the LP25 EVs from two methods that induced RAW 264.7 macrophage polarization were observed (**Supplementary Figures S3A, B**).

### 3.4 Increased expression of M2 macrophage markers in LPS-stimulated RAW264.7 cells treated with LP25 EVs

**Figure 5:**
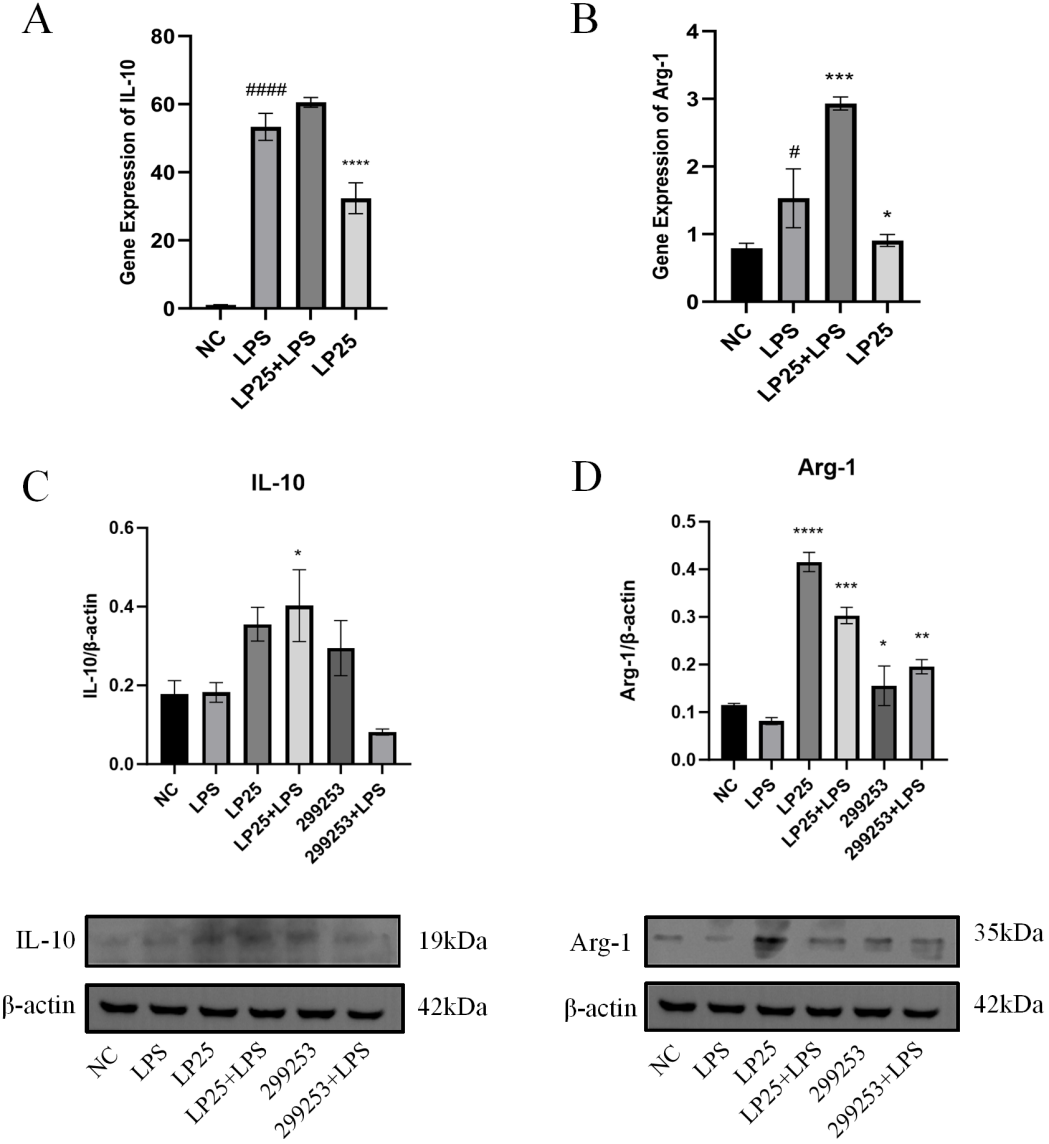
LP25 EVs modulates the expression of M2 macrophage markers. **(A, B)** qPCR detection of IL-10 and Arg-1 expression after 18 hours of LPS(1μg/ml)treatment or LPS(1μg/ml)/LP25 EVs(240ng/ml)treatment; **(C, D)** Representative Western blot and average quantitative data from three independent experiments of IL-10, Arg-1, and β-actin expression. Error bars represent mean ± SD from three independent experiments. ^#^ *p*<0.05, ^####^ *p*<0.0001 indicates a significant difference from NC, NC indicate LPS-untreated cells; *p < 0.05,**p < 0.01, ***p < 0.001, ****p < 0.0001 indicate significant differences from the LPS alone.

To investigate whether LP25 EVs could regulate the polarization of LPS-stimulated RAW264.7 cells, we assessed the mRNA and protein expression of M2 phenotype macrophages using qPCR and Western blotting. As shown in **Figure 5**, LP25 EVs treatment for 18 hours increased the levels of M2 phenotype markers IL-10 and Arg-1 in LPS-stimulated RAW264.7 cells (**Figures 5A, B**). The protein expression of IL-10 and Arg-1 was also increased, consistent with the qPCR results (**Figures 5C, D**).

### 3.5 LP25 EVs treatment reduces the expression of M1 macrophage markers in peritoneal lavage cells from C57BL/6 mice injected intraperitoneally with LPS

**Figure 6:**
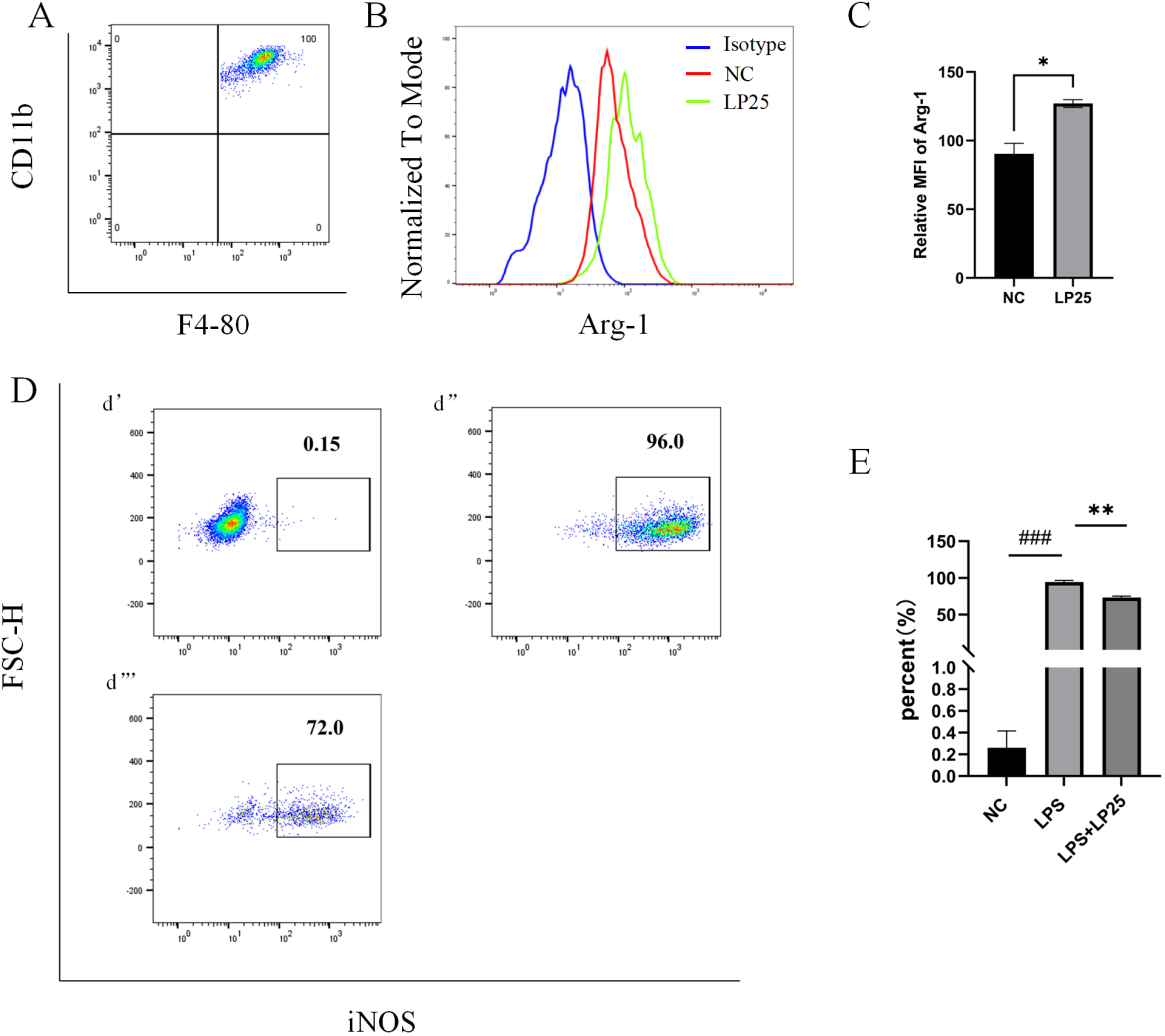
Expression of iNOS and Arg-1 in mouse peritoneal macrophages after intraperitoneal injection of LPS with or without LP25 EVs treatment. C57BL/6 mice received an intraperitoneal injection of 200 μl of LP25 EVs (5 mg/mL). After 8 hours, peritoneal cells were harvested by washing the peritoneal cavity with PBS. The collected cells polarization level were detected by Flow Cytometry. **(A)** Density plot of F4/8-CD11b macrophages; **(B, C)** Representative flow cytometry histograms and average relative mean fluorescence intensity (MFI) of Arg-1 at 8 hours from two independent experiments; **(D)** Expression of iNOS in peritoneal lavage cells (d’-d’’’ represent NC, LPS, LPS+LP25 EVs groups respectively); **(E)** Percentage of iNOS at 8 hours from two independent experiments. Error bars represent mean ± SD from two independent experiments. ^###^ *p*<0.001 indicates a significant difference from NC,NC indicate LPS-untreated cells, *p < 0.05, **p < 0.01 indicate significant differences from the LPS alone.

Arg-1 serves as a marker for the M2 phenotype of macrophage (9). Flow cytometry results showed that the Arg-1 expression level was significantly upregulated in mice intraperitoneally injected with 1 mg/ml LP25 EVs compared to untreated group at 8 hours (**Figures 6A–C**).Simultaneous intraperitoneal injection of LP25 EVs with LPS (2.5 mg/kg) resulted in a downward trend of iNOS compared to the LPS group at 8 hours (**Figures 6D, E**). These data collectively indicate that LP25 EVs can modulate the polarization of RAW 264.7 cells stimulated by LPS, promoting the formation of the M2 phenotype and reducing the expression of the M1 marker iNOS.

## 4 Discussion

In this study, we screened various strains of *L. plantarum* for anti-inflammatory properties, focusing on changes in RAW 264.7 macrophage morphology and IL-10 expression after LPS stimulation. The LP25 strain was identified as having significant anti-inflammatory activity. Further *in vitro* analysis demonstrated that treatment with LPS and LP25-derived extracellular vesicles (LEVs) led to decreased expression of M1 macrophage markers (TNF-α, IL-6, iNOS, and CD86) and increased expression of M2 macrophage markers (Arg-1 and IL-10). These findings were corroborated by *in vivo* experiments, which showed higher Arg-1 expression in mice treated with LEVs compared to control groups. This study provides compelling evidence that LEVs can modulate macrophage polarization, promoting anti-inflammatory M2 phenotypes and reducing inflammation.

Macrophages play a critical role in innate immune surveillance, and their proper activation in response to environmental cues is crucial for tissue homeostasis (6). Macrophages exhibit pleiotropy and functional diversity, capable of altering their activation state (M1 or M2) in response to growth factors and external stimuli, such as cytokines, chemokines, microorganisms, and microbial products (16). Probiotics have been shown to modulate inflammation in a strain-specific manner (17). As a representative probiotic species, *L. plantarum* is commonly used in studies of anti-inflammatory activity (18). Different strains of *L. plantarum* have demonstrated significant anti-inflammatory and immunomodulatory effects. Research indicates that *L. plantarum* A106 enhances antioxidant and anti-inflammatory activities by improving mitochondrial function, significantly reducing TNF-α and IL-6 levels in LPS-induced RAW 264.7 macrophages, and regulating the expression of related genes (19). In a rat model of peripheral neuropathy, *L. plantarum* mitigates neuropathic pain by promoting macrophage M2 polarization, increasing short-chain fatty acid (SCFA) levels, and enhancing the release of anti-inflammatory cytokines (20). Another strain, *L. plantarum* MWFLp-182, improves D-galactose-induced cognitive impairment by modulating the gut-brain axis, increasing the expression of anti-inflammatory cytokines, and decreasing the expression of pro-inflammatory cytokines (21). Additionally, *L. plantarum* T10 enhances intestinal barrier function both *in vitro* and *in vivo* through its exopolysaccharide products, significantly alleviating DSS-induced colitis (22). Moreover, postbiotic fractions of *L. plantarum* 299v show remarkable immunomodulatory effects, effectively activating the anti-inflammatory cytokine IL-10 and modulating the IL-18-related response, thereby controlling inflammation and protecting the intestinal epithelial barrier (23). These studies suggest that different strains of *Lactiplantibacillus plantarum* have potential clinical applications in anti-inflammatory and immunomodulatory therapies. Recently, EVs derived from Gram-positive bacteria have gained increasing attention due to their impact on human health (24).This study reports that EVs derived from *L. plantarum* LP25 can reduce the expression of inflammatory cytokines and proteins in LPS-stimulated RAW 264.7 macrophages and induce the differentiation of these cells toward the M2 phenotype.

The anti-inflammatory activity of EVs is primarily observed in non-pathogenic species like probiotics. Choi et al. (25) found that EVs from *Lactobacillus paracasei* reduced the expression of LPS-induced cytokines (IL-1α, IL-1β, IL-2, and TNF-α). Rezende et al. (26) demonstrated that EVs from the Gram-positive probiotic *Propionibacterium freudenreichii* regulated NF-κB transcription factor activity and IL-8 release, alleviating inflammation both *in vitro* and *in vivo*. Additionally, lipoteichoic acid from *Lactobacillus reuteri* DSMZ 8533 inhibited induced inflammation in macrophage-like cells (27). Other studies have found that peptidoglycans (PGN) from *L. plantarum* strains CAU1054, CAU1055, CAU1064, and CAU1301 significantly inhibited LPS-induced inflammation in RAW 264.7 mouse macrophages by regulating iNOS, COX-2, IL-6, and TNF-α expression (28). These findings highlight the significant interactions between probiotics and the host immune system. The findings of this study have significant implications for the development of probiotic therapies. The ability of LEVs from LP25 to modulate macrophage polarization and reduce inflammation positions them as a promising candidate for anti-inflammatory probiotic treatments. Compared to conventional anti-inflammatory drugs, probiotics and their derivatives like LEVs offer a natural, potentially safer alternative with fewer side effects. Moreover, the targeted modulation of immune responses by LEVs could provide therapeutic benefits for a range of inflammatory conditions, including inflammatory bowel disease, arthritis, and skin inflammation.

The mechanism by which LEVs induce macrophage polarization remains unclear. Comprehensive analysis of macrophage-related cell markers, chemokines, and cytokine gene expression revealed that LEV-treated THP1 macrophages primarily differentiated into M2 macrophages, particularly the M2b subtype, which is known for its strong immunoregulatory and anti-inflammatory properties, thus modulating the breadth and depth of immune responses (29). Extracellular vesicles (EVs) from *L. plantarum* species, have shown potential in modulating immune responses through various mechanisms, including the regulation of endoplasmic reticulum (ER) stress pathways. Research indicates that *L. paracasei*-derived EVs can attenuate inflammation by augmenting the ER stress pathway, leading to a reduction in pro-inflammatory cytokines and an increase in anti-inflammatory cytokines like IL-10 (25). TRIM29, an E3 ubiquitin ligase, plays a critical role in regulating immune responses by modulating the activation of macrophages and the production of pro-inflammatory cytokines (30). Furthermore, PERK, a key protein in the ER stress response, is crucial for the metabolic reprogramming of M2 macrophages, promoting their immunosuppressive functions (31). TRIM29 has been shown to interact with PERK, promoting its stability and enhancing ER stress responses, which are essential for the polarization and function of M2 macrophages (32).The interplay between TRIM29 and PERK-mediated signaling pathways suggests that L. plantarum-derived EVs could potentially regulate TRIM29 expression, thereby influencing PERK activity and promoting M2 macrophage polarization. This mechanism would contribute to the anti-inflammatory effects observed with these EVs, making them a promising therapeutic approach for inflammatory diseases.

In conclusion, our study demonstrates that extracellular vesicles (EVs) from the LP25 strain of Lactiplantibacillus plantarum effectively modulate macrophage polarization, promoting an anti-inflammatory M2 phenotype and reducing inflammation. These findings highlight the significant potential of these EVs as anti-inflammatory agents in probiotic therapies. By enhancing our understanding of the underlying mechanisms, particularly involving TRIM29 and PERK-mediated ER stress signaling pathways, future research can pave the way for novel, natural treatments for various inflammatory diseases, including inflammatory bowel disease, arthritis, and skin inflammation.

## Data Availability

The datasets presented in this study can be found in online repositories. The names of the repository/repositories and accession number(s) can be found in the article/**Supplementary Material**.
