# Supplementary material for: Extracellular vesicles from a novel Lactiplantibacillus plantarum strain suppress inflammation and promote M2 macrophage polarization
Source: Front Immunol. 2024 Aug 23;15:1459213. doi: 10.3389/fimmu.2024.1459213 (PMC11377267; doi:10.3389/fimmu.2024.1459213)
Supplement: Supplementary file 1 [file DataSheet1.docx]

Supplementary Material

# Biological Characteristics Analysis of *Lactiplantibacillus plantarum* LP25

The strain can grow in a microaerobic environment with 5.0% CO2, and it also grows well under anaerobic and aerobic conditions. It grows within a pH range of 2.0 to 10.0, with an optimal pH of 7.0 (Supplementary Figure S1b). The strain grows within a temperature range of 10 to 45℃, with optimal growth at 37°C (Supplementary Figure S1c). The optimal salt concentration for growth is 0%, and it can grow in salt concentrations up to 5% (Supplementary Figure S1d). Genomic DNA of strain LP25 was extracted using a bacterial genome DNA extraction kit, and the extracted DNA was amplified using 16S rRNA universal primers. The PCR product was subjected to agarose gel electrophoresis and gel extraction, and the product was sent to Sangon Biotech (Shanghai) Co., Ltd. for sequencing. The 16S rRNA sequence obtained from gene prediction was compared with the NCBI 16S database using Blast, with parameters set to identify > 95%. The top 30 16S rRNA sequences with the highest identity (or all if fewer than 30) were selected and subjected to multiple sequence alignment using mafft software and trimmed, followed by construction of a phylogenetic tree using FastTree software (Supplementary Figure S1e), showing LP25 homology with Lactobacillus plantarum. Based on the homology gene results, a core gene phylogenetic tree was constructed using the single-copy core gene set with Neighbour-joining clustering (Supplementary Figure S1f). The 16S rRNA gene tree and core genome tree showed similarity.


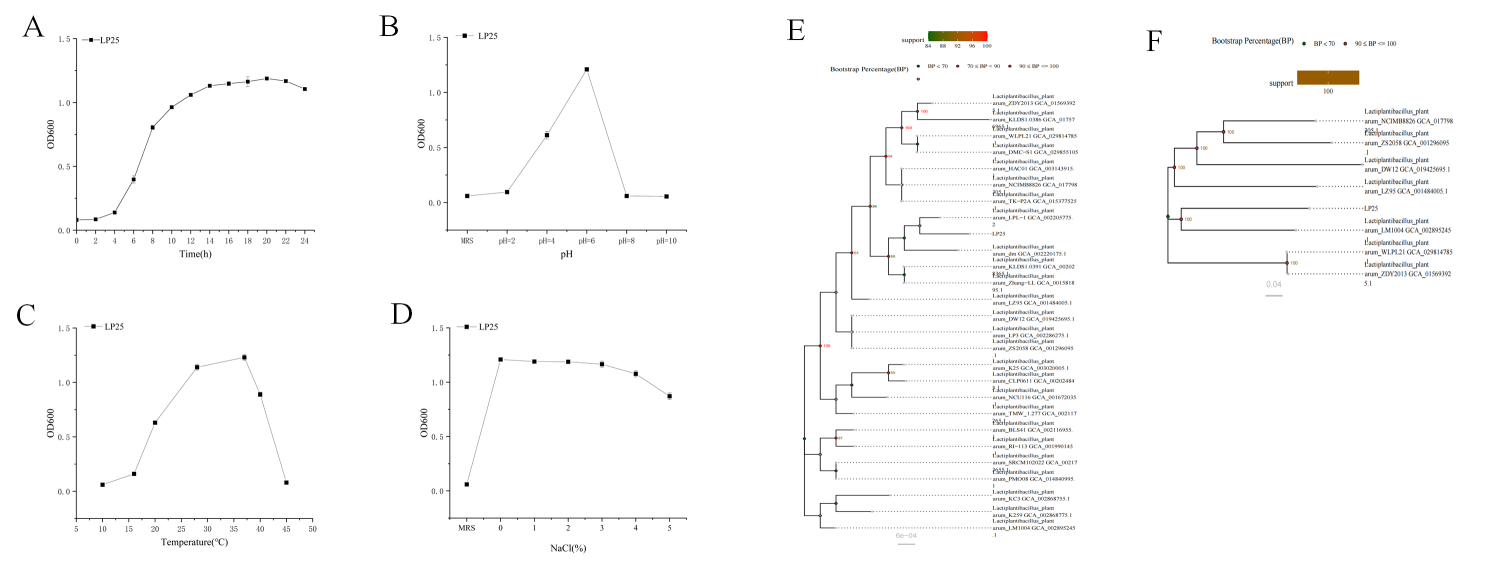


**Supplementary Figure S1** Growth Characteristics of LP25.(A): Growth curve of LP25; (B): Effect of pH on LP25 growth; (C): Effect of temperature on LP25 growth; (D): Effect of salt concentration on LP25 growth; (E): LP25 16S rRNA gene tree; (F): LP25 core gene tree.

# Extracellular Vesicles Mitigate LPS-Induced Apoptosis in RAW 264.7 Cells

Using *L. plantarum* 299253 EVs (240 μg/ml) as a control, RAW 264.7 cells were treated with LEV (240 μg/ml) and 299253 EVs separately, while equal amounts of LEV and 299253 EVs were added to LPS-induced cells .After an 18-hour incubation,RAW 264.7 cells were washed once with PBS, and with 4% paraformaldehyde fixed for 30 minutes.PBS washing cells again, and then add in 150 ul containing 0.1% of Triton X-100 PBS, incubation at room temperature for 15 minutes,50μl of TUNEL assay solution was added to the samples and incubated at 37 °C in the dark for 60 minutes.Finally, the DAPI at room temperature away from light incubation for 5 minutes.Cells treated with LEV did not show apoptosis and LEV was found to attenuate LPS-induced apoptosis levels (Supplementary Figure S2).


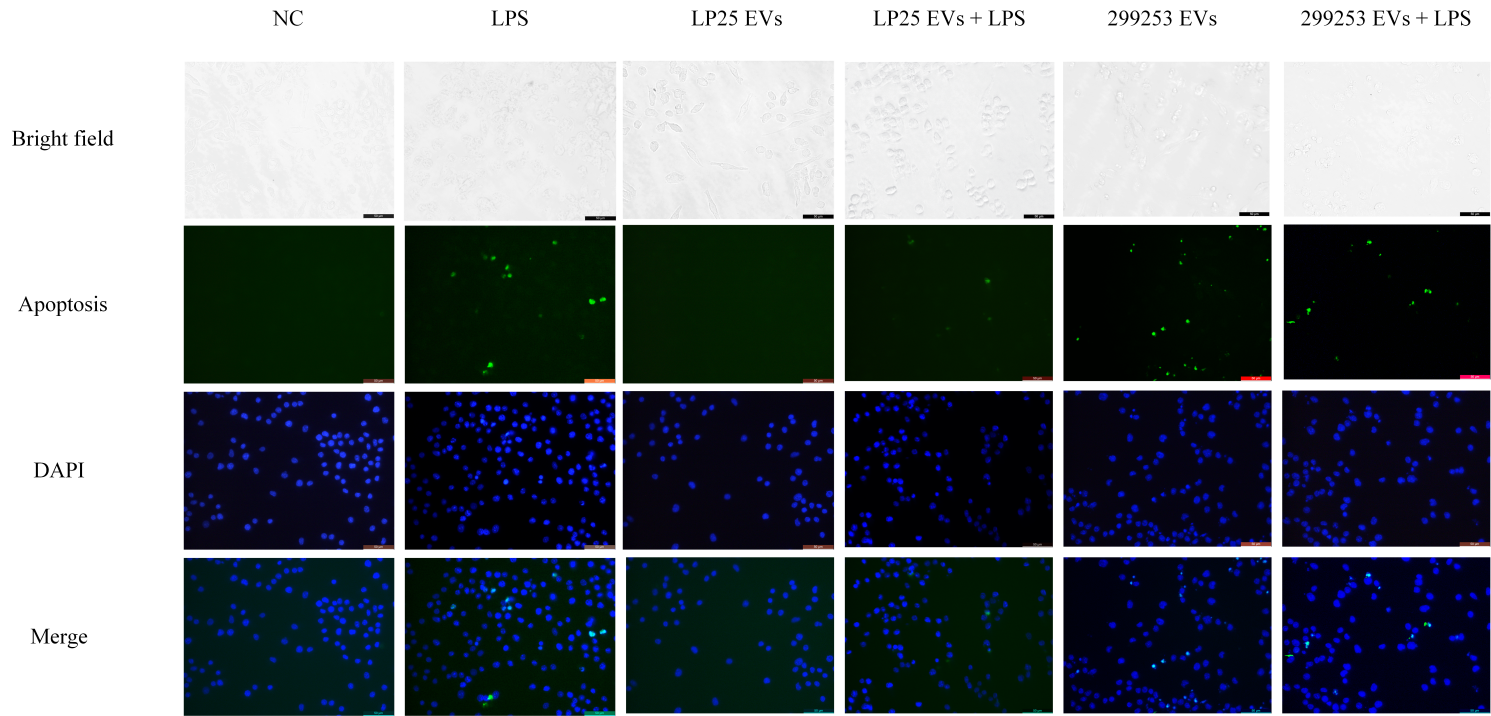


**Supplementary Figure S2** Effects of LP25 EVS and 299253 EVs on LPS-induced apoptosis in RAW 264.7 cells. RAW 264.7 cells were treated with LEV (240 μg/ml) and 299253 EVs (240 μg/ml), either alone or in combination with LPS. After 18 hours of incubation, cells were fixed and subjected to TUNEL assay for apoptosis detection, followed by DAPI staining for nuclear visualization.Scale bars represent 20 μm.

# Comparison of Exosomes Obtained by PEG Precipitation and Ultracentrifugation

RAW 264.7 macrophages treated with LPS stimulation and exosomes (240 μg/ml) obtained from PEG precipitation and ultracentrifugation were analyzed for the expression of M2 polarization marker Arg-1 protein, with no significant difference observed.


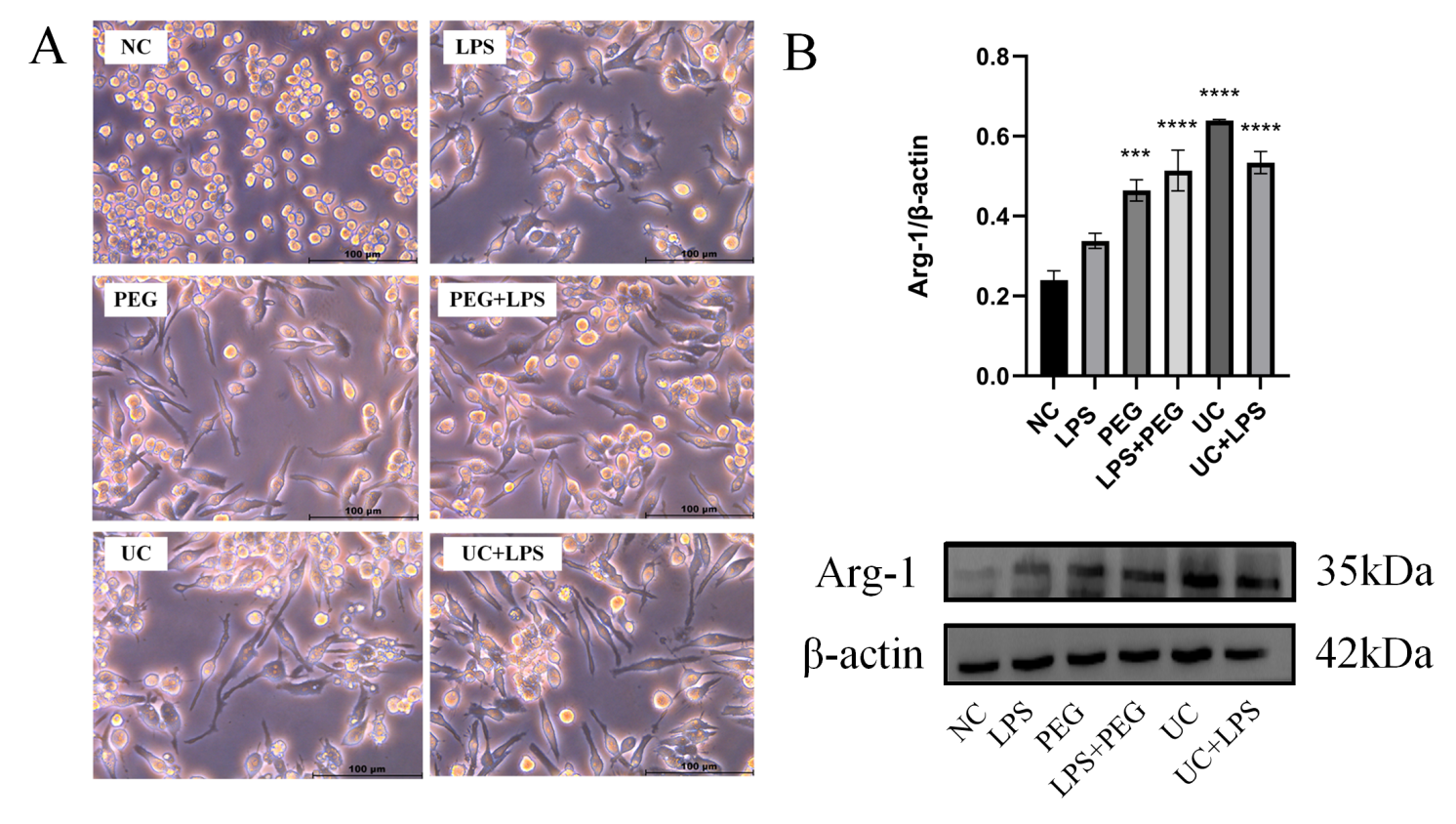


**Supplementary Figure S3** RAW 264.7 macrophages treated with exosomes from PEG precipitation and ultracentrifugation.(A): Images of RAW 264.7 macrophages treated with LEV obtained by PEG precipitation and ultracentrifugation (scale bar = 100 μm); (B)Western blot analysis of M2 polarization protein Arg-1 in LPS-stimulated RAW 264.7 macrophages treated with EVs extracted by PEG precipitation and ultracentrifugation. Compared with the LPS group, ***p < 0.001, ****p< 0.0001.

**
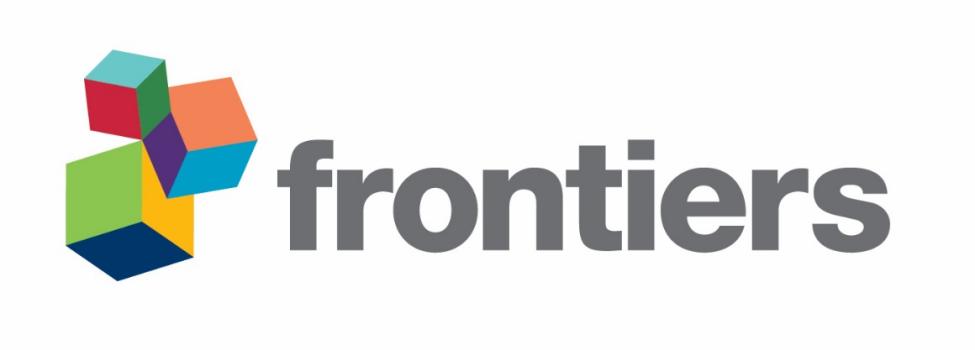
**
